# A Neurosurgical Golf Injury

**DOI:** 10.4137/ccrep.s736

**Published:** 2008-05-22

**Authors:** R. Al-Mahfoudh, S. Clark, J. Kandasamy, P. May

**Affiliations:** Department of Neurosurgery, the Walton Centre, Liverpool.

**Keywords:** golf ball, extradural haematoma, golf injuries

## Introduction

Being one of the few activities that people of all ages and skill level can play, golf has increased in popularity. Consequently golf-related injuries have been notably increasing over the past few years. This particularly occurs in the paediatric age group.^1^ Head injury in this sport is most likely to be caused by golf clubs, however there have been reported cases in the literature of golf ball head injuries.^2^

We present a case of an extradural haematoma secondary to a golf ball injury. To our knowledge there has not been a report of an extradural haematoma secondary to a golf ball injury (pubmed search). A high index of suspicion combined with early investigation and prompt management are essential in managing this type of injury.

## Case Report

A 16 year old gentleman was in a golf bunker, when he was hit by a golf ball on the left temporal aspect of his skull. The flight distance of the ball can be roughly estimated as it took the “offending player!” 2 minutes to reach this young gentleman to check on his wellbeing. As he had no symptoms initially he continued to play. Following completion of the game (30 min later), he returned home. Around 15 minutes after arriving home he felt unwell and then developed right sided weakness, expressive dysphasia and blurred vision in the right eye. His parents subsequently took him to the emergency department.

On examination, the patient was haemodynamically stable. Neurological examination revealed a GCS of 13/15 (E3 M6 V4), right 6th nerve palsy and a right sided hemiparesis (grade 4/5).

**Figure 1:**
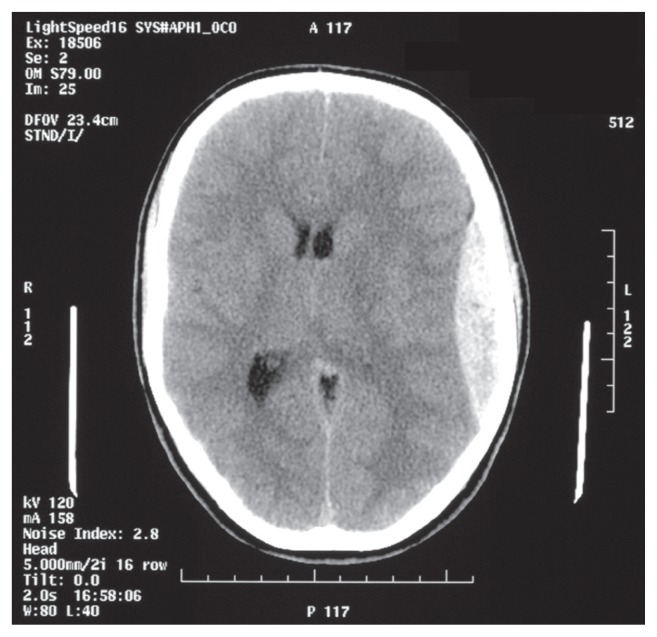
CT scan demonstrating left extradural haematoma.

A CT scan was organized and this revealed a left tempero-parietal extradural haematoma with mass effect and an associated temporal fracture (Fig. 1).

The patient was transferred to our neurosurgical department where an evacuation of the haematoma was successfully undertaken and haemostasis achieved. The post-operative CT scan demonstrated satisfactory removal of the clot.

Post operatively the hemiparesis and sixth nerve palsy improved. Neurological examination revealed no deficit with a GCS of 15/15. The patients’ recovery was uneventful and he was discharged home four days later.

## Discussion

There are less than 20 case reports of golf related head injuries in the literature mainly in the paediatric age groups reflecting an emerging pattern of sport related trauma. Our review of the literature did not reveal any reports of an extradural haematoma resulting directly from a golf ball.

Extradural haematomas (EDHs) usually occur (85% of cases) as a result of linear squamous temporal skull fractures with laceration of a branch of the underlying middle meningeal artery. In the remainder of cases the bleeding is usually from the middle meningeal vein or dural sinuses. EDH represents 1% of head trauma admissions. It is more likely to occur in young adults as the dura is easily stripped off the inner table of the skull.

The classical presentation is a brief post traumatic loss of consciousness followed by a lucid interval and later a decreasing GCS, contralateral hemiparesis and ipsilateral papillary dilatation.

Dysphasia or aphasia may occur with left dominant hemispheric lesions. Ipsilateral papillary dilatation occurs due to downward displacement of the uncus compressing the brainstem and ipsilateral third nerve. Contralateral hemiparesis occurs due to compression of the ipsilateral corticospinal tracts in the crus cerebri. Abducens nerve palsy may occur due to increased intra cranial pressure and maybe a false localizing sign due to its long intracranial course making it more sensitive to raised pressure.

With regards to management of EDHs, a high index of suspicion, prompt diagnosis and treatment are required, in order to prevent neurological deterioration. The classical CT appearance is a biconvex hyper density adjacent to the inner table of the skull.

Small asymptomatic EDHs may be treated conservatively with close monitoring of the patients neurological status initially and followed up with interval scans.

Surgery should be considered for any symptomatic EDH, or acute asymptomatic EDH >1 cm in its thickest diameter.

## Conclusion

Golf-related injuries have been increasing with an increasing popularity of the sport. These are mostly overuse injuries associated with the back, neck and shoulder.^3^ Nevertheless golf has been identified as a potential cause of head injuries.^4^ Injuries from ball strikes remain to be rare. Our case demonstrates a more serious injury (extradural haematoma) which is potentially fatal. Prevention of such injuries in the future through the use of safety measures including player education, ensuring no one is within range of the golfer’s club or ball and possibly the use of helmets for the paediatric age group in addition to close parental supervision is recommended.

## Figures and Tables

**Figure 2:**
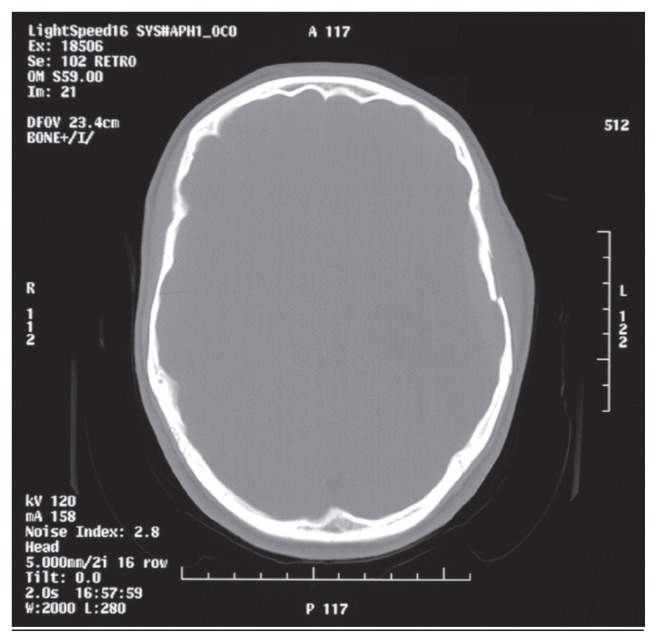
CT scan (bone window) demonstrating the temporal bone fracture.

**Figure 3:**
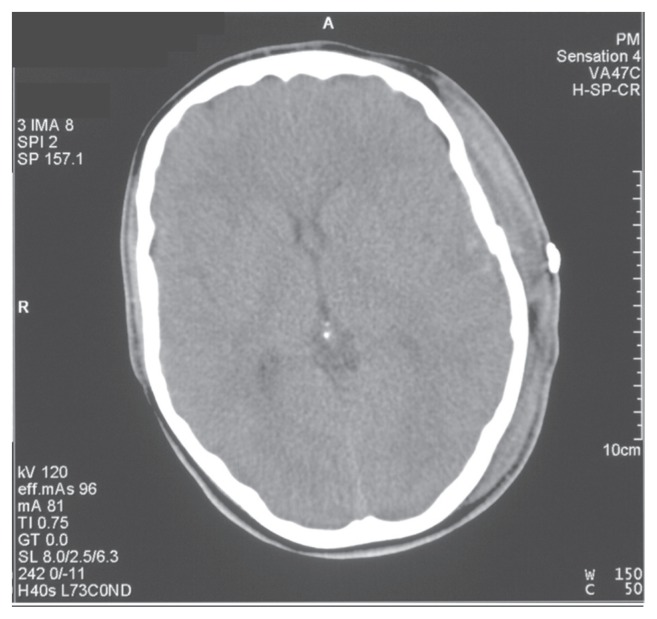
Post operative CT scan, the haematoma has been evacuated.

## References

[1] Rahimi SY, Singh H, Yeh DJ, Shaver EG, Flannery AM, Lee MR (2005). Golf-associated head injury in the pediatric population: a common sports injury. Journal of Neurosurgery.

[2] Macgregor DM (2002). Golf related head injuries in children. Emergency Medicine Journal.

[3] McHardy A, Pollard H, Luo K (2006). Golf injuries: a review of the literature. Sports Medicine.

[4] Lindsay KW, McLatchie G, Jennett B (1980). Serious head injury in sport. British Medical Journal.

